# The Preventive Role of Hydrogen-Rich Water in Thioacetamide-Induced Cholangiofibrosis in Rat Assessed by Automated Histological Classification

**DOI:** 10.3389/fphar.2021.632045

**Published:** 2021-08-20

**Authors:** Chaofu Li, Xing Zhao, Xiaoqiang Gu, Ying Chen, Guanzhen Yu

**Affiliations:** ^1^ Department of Oncology, Longhua Hospital Affiliated to Shanghai University of Traditional Chinese Medicine, Shanghai, China; ^2^ Department of Oncology, Fourth Affiliated Hospital of Guangxi Medical University, Liuzhou, China; ^3^ Department of Oncology, Xiangyang Central Hospital, Affiliated Hospital of Hubei University of Arts and Science, Xiangyang, China; ^4^ Department of Pathology, Changhai Hospital, Shanghai, China; ^5^ Shanghai Key Laboratory of Multidimensional Information Processing, East China Normal University, Shanghai, China

**Keywords:** H_2_, cancer prevention, artificial intelligence, quality of life, cholangiofibrosis

## Abstract

**Background:** Cholangiofibrosis is a controversial intrahepatic cholangial lesion that precedes the development of cholangiocarcinoma. Here, we demonstrate that molecular hydrogen (H_2_) can be used to effectively prevent cholangiofibrosis.

**Methods:** The safety and quality of life (QOL) of rats was firstly evaluated. H_2_ was administered to rats subjected to thioacetamide (TAA)-induced cholangiofibrosis throughout the whole process. Then, rats were administrated with TAA for 3 months and then followed by H_2_ intervention. Rat livers were harvested and assessed by light microscopy and convolutional neural network. RNA-seq was performed to analyze the genetic changes in these animal models.

**Results:** Continuous use of H_2_-rich water was safe and improved QOL.The incidence and average number of cholangiofibrosis in the liver were higher in the TAA group (100%, 12.0 ± 10.07) than that in the H_2_ group (57.1%, 2.86 ± 5.43). The AI algorithm revealed higher Alesion/Aliver in the TAA group (19.6% ± 9.01) than that in the H_2_ group (7.54% ± 11.0). RNA-seq analysis revealed that H_2_ results in a decline in glycolysis. Moreover, in the third experiment, the incidence of microscopic or suspicious tumors and the ratio of liver lesions was decreased after long-term use of H_2_ (12.5%, 0.57% ± 0.45) compared with untreated group (100%, 0.98% ± 0.73). A number of intestinal microbiota was changed after H_2_ usage, including clostridiaceae_1, ruminococcus, turicibacter, coriobacteriales, actinobacteria, and firmicutes_bacterium.

**Conclusion:** Hydrogen-rich water protects against liver injury and cholangiofibrosis and improved quality of life partially through regulating the composition of intestinal flora.

## Introduction

Cancer incidence and mortality rates in China have been rapidly increasing (Bode et al., 2016). Not surprisingly, the incidence of traditional Chinese cancers, such as liver, stomach, esophageal, nasopharyngeal, and cervical cancers, remains high, while the incidence and mortality of cancers associated with unhealthy lifestyles and pollution, such as lung, breast, colon, and prostate cancers, have dramatically increased over the past decade (Chen et al., 2016). The most commonly accepted reasons for this increase are rapid industrialization and urbanization accompanied by drastic alterations in lifestyle and the environment. The endless pursuit of wealth has led to the irrevesible deterioration of the enviroment at a rapid pace, which correlates with increases in cancer incidence and mortality. The existence of “cancer villages” (i.e., cancer clusters) offers evidence of a causal link between cancer incidence and environmental carcinogens (Lu et al., 2015). Unfortunately, no effective strategies are available for the protection or reversal of environmental pollution. Therefore, we require an alternative strategy of "preventing the preventable" (Molokhia and Perkins, 2008).

Chemoprevention refers to the use of natural, synthetic, or biological agents to avoid, delay, reverse, or inhibit cancer initiation and tumor progression (Walczak et al., 2017). An ideal chemopreventive agent is a compound that can be administered over a long period of time to individuals with a high risk of develping cancer without causing severe side effects. In observational studies and clinical trials, drugs such as aspirin, metformin, and tamoxifen decrease cancer incidence with limited side effects (Rothwell et al., 2012; Chan, 2016; Rondanina et al., 2017). Long-term aspirin use reduces the incidence and mortality of cancer, particularly in colorectal carcinoma (Rothwell et al., 2010) and in cholangiocarcinoma (Itoh et al., 2011). Similarly, a recent clinical trial suggested that low-dose metformin could reduce the prevalence and number of metachronous adenomas and polyps after polypectomy (Higurashi et al., 2016). However, the harmful effects of aspirin (gastrointestinal bleeding) and metformin (gastrointestinal disturbance) are potential side effects that may not be acceptable in standard regimens or even in off-label use for cancer prevention. In this study, we aimed to develop a safer, more natural, and more easily acceptable alternative modality to “prevent the preventable”.

Molecular hydrogen (H_2_) has potential as a “novel” antioxidant in preventive and therapeutic applications (Ohta, 2011). The positive therapeutic effects of H_2_ on a rat model of cerebral infarction was reported in *Nature Medicine* in 2007 (Ohsawa et al., 2007). Moreover, an overwhelming number of studies have explored the therapeutic effects of H_2_ in a wide range of disease models, human diseases, and treatment-associated pathologies (Ichihara et al., 2015). Most of these diseases are associated with oxidative stress-associated diseases and inflammatory diseases. The anti-tumor effects of H_2_ have long been investigated. In 1975, Dole et al. first reported in *Science* that a solution containing 97.5% hydrogen reduced the growth of the squamous cell carcinomas in nude mice (Dole et al., 1975). Recent studies revealed that hydrogen could inhibit tumor cell proliferation and tumor angiogenesis while reducing the side effects of radiation and chemotherapy (Saitoh et al., 2008; Ye et al., 2008; Zhao et al., 2011; Li et al., 2019; Runtuwene et al., 2015). These data support the chemopreventive and therapeutic potential of H_2_ in human solid cancers. In the preliminary study, we have proved that long-term use of H_2_–rich water could reduced 42.9% incidence of cholangiofibrosis (misdiagnosed as cholangiocarcinoma in various studies (Yeh et al., 2004; Dwyer et al., 2021) in a rat model induced by thioacetamide (TAA) (Li et al., 2019). Here, we further investigated these specimens using an AI algorithm and explored its possible mechanisms.

## Materials and Methods

### Animal Models

All experimental procedures were approved by the Experimental Animal Ethics Committee of Dongfang Hospital Affiliated with Tongji University in Shanghai, China. Rats were housed as previously described (Yeh et al., 2004). Three experiments were performed in this study. The first enrolled 20 adult male Sprague-Dawley (SD) rats, which were divided into two groups: regular water group and H_2_-rich water daily. These rats were not adminstrated with TAA. This experiment was used to decide the safety of long-term use of H_2_-rich water and whether H_2_-rich water could improve the quality of life (Supplementary Figure S1).

The second enrolled 38 adult male Sprague-Dawley (SD) rats (300–370 g), which were administrated with TAA with or without hydrogen-rich water for 5 months. These rats were divided into two groups: TAA (*n* = 19) and TAA + H_2_ (n = 19). All rats were administered 300 mg/L TAA in their drinking water daily until they were sacrificed as previously described (Li et al., 2019). In addition, the experimental groups were intragastrically (ig) administered 1.8 mg/L hydrogen-rich water daily (final volume: 2 ml) (Shanghai Purplebox Bio-Tec Co., Ltd.), whereas the control group was administered 2 ml of regular water ig. 8 weeks after treatment, three animals per group were sacrificed monthly, and the livers were harvested to examine the oncogenic effects of TAA and the preventive effects of H_2_. The animals were weighed weekly.

The third enrolled 30 adult male Sprague-Dawley (SD) rats. These rats were feed with the same TAA water for 3 months and stopped, which were then divided into two groups: regular water group and H_2_-rich water daily. This experiment was used to detect whether H_2_-rich water could interrupt the progression of CCA.

### Harvesting Procedure and Histopathological Evaluation of the Livers

At the indicated time, all animals were sacrificed with CO2 flow (10–30% of the cage volume per minute). Following a midline laparotomy, all lobes of the liver were assessed for disease and then excised. The gross morphology of the liver was carefully examined and scored. The number of lesions on the surface of the liver was determined. The inferior vena cava was used to draw blood samples to test for liver function and other physiological readouts. The liver was washed with water and cut into 3–5 mm sections; half of each liver sample was stored at −80°C, and the other half was fixed in 4% paraformaldehyde. The number of lesions in each tissue section was also counted.

To evaluate the lesions objectively and quantitatively, we developed an automated lesion detection framework based on convolutional neural networks (CNNs) for the detection of liver lesions. The tissue regions were first extracted from whole slide images (WSIs) using Otsu threshold algorithm followed by morphological operations, and the corresponding tissue areas, A_tissue_, were calculated. Subsequently, the automated lesion detection framework based on deep learning was proposed to detect and localize the fibrosis and cancer regions from the tissue regions. The framework consists of a patch-level classification and a post-processing process to generate the lesion likelihood map for the WSI. We run the patch-wise CNN classifier across the tissue region in a sliding window manner. For each patch, the classifier was used to give a lesion-probability. The probability was then accumulated to get the likelihood map for the WSI. After that, the likelihood map was normalized so that each pixel has a continuous likelihood ranged from 0 to 1. To obtain the lesion areas, A_lesion_, the likelihood map was thresholded at 0.9 to get rid of the low likelihood areas. Given the whole tissue area, A_tissue_, and the lesion area, A_lesion_, of the WSI, we can then calculated the ratio by ratio = Alesion/Atissue.

### RNA-Seq

Sequencing and analysis were performed using normal muscle or intestinal tissues, as well as lesion-harboring livers, for four rats administered TAA only and four rats treated with TAA + H_2_ from the first experiment at Shanghai Oe Biotech Co. Ltd. Illumina HiScan™ 2,500 was used as the platform for RNA-seq. First, total RNA was extracted using TRIzol reagent (Invitrogen) and purified using an RNeasy Mini Kit (Qiagen). RNA-seq libraries were prepared according to instructions included in the standard Illumina library preparation kit. Sequencing quality of the raw data was assessed using both FastQC and NGS QC TOOLKIT v2.3.3, after which contaminating adaptor sequences were removed using FastqMcf (v1.4). Paired reads were aligned to the B73 reference genome (RefGen_v3) and the reference gene model dataset (FGS 5b) using TopHat/Bowtie2. The Refseq genes values were normalized as fragments per kilobase of an exon model per million mapped reads (FPKM). Quantitative gene expression analysis was performed using the Cufflinks package, and differential expression analysis was performed using the DESeq software package.

### Immunohistochemistry

The skins from the rats in the first experiment and the livers from the rats in the second experiment were collected, fixed in neutral buffered formalin and embedded in paraffin. 4 μm slides were prepared for immunohistochemical staining with CK7 (A4357, ABclonal-Bio, Wuhan, China), CK19 (A0247, ABclonal-Bio, Wuhan, China), α-SMA (AM-0051, Maixin-Bio, Fuzhou, China), Ki67 (Kit-0005, Maixin-Bio, Fuzhou, China), AKR1C1 (PB1091, Boster Biological Technology, Wuhan, China).

#### Intestinal Microbiota of Rats From the Second Experiment Was Analyzed by High-Throughput Sequencing

Stool samples were collected from colon of rats from the second experiment and stored at −80°C before DNA isolation using the TopTaq DNA Polymerase Kit (Transgen, China). The targeted V3-V4 hypervariable region was amplified with the primer pair F: Illumina adapter sequence 1 + CCTACGGGNGGCWGCAG and primer pair R: Illumina adapter sequence 2 + GACTACHVGGGTATCTAATCC. Amplicons from all samples were multiplexed and paired-end sequenced on an Illumina Miseq at Genesky Biotechnologies Inc., Shanghai, China, according to a published dual-indexing protocol (Xie et al., 2017).

#### Data Preprocessing and Analysis

All datasets were 16S rRNA gene amplicon sequencing data from the gut microbial communities and low-quality reads (Q < 20) were firstly to be removed from raw data. Nonspecific amplicons, reads with an error rate >2, singletons, and chimeric sequences were then removed by USEARCH to obtain clean data. Qualified sequences were clustered into operational taxonomic unites (OTUs) at 97% similarity with UPARSE (Edgar, 2013). Representative sequences of OTUs were classified taxonomically based on the Ribosomal Database Project (RDP) database. We calculated α- and β-diversities R using software packages (V2.15.3, http://www.r-project.org/). We performed cluster analysis using the Bray−Curtis dissimilarity index and the unweighted pair-group method with arithmetic means (UPGMA) linkage method. Canonical correspondence analysis (CCA) was performed for the analysis of the contributions of diCQAs to the microbial composition. The difference in microbial communities of each group was analyzed by linear discriminative analysis (LDA) effect (Xie et al., 2017; Jiao et al., 2018a).

### Statistics

All statistical analyses were performed using SPSS and GraphPad Prism (version 5.0) software. Categorical data were analyzed using χ2 tests. Differences between two groups were determined using a two-tailed Student’s t-test. Comparison among three groups should be evaluated with ANOVA followed by post hoc tests. A *p* value less than 0.05 was considered statistically significant.

## Results

### Safety and Quality of Life for Long-Term Use of H_2_-Rich Water

**FIGURE 1:**
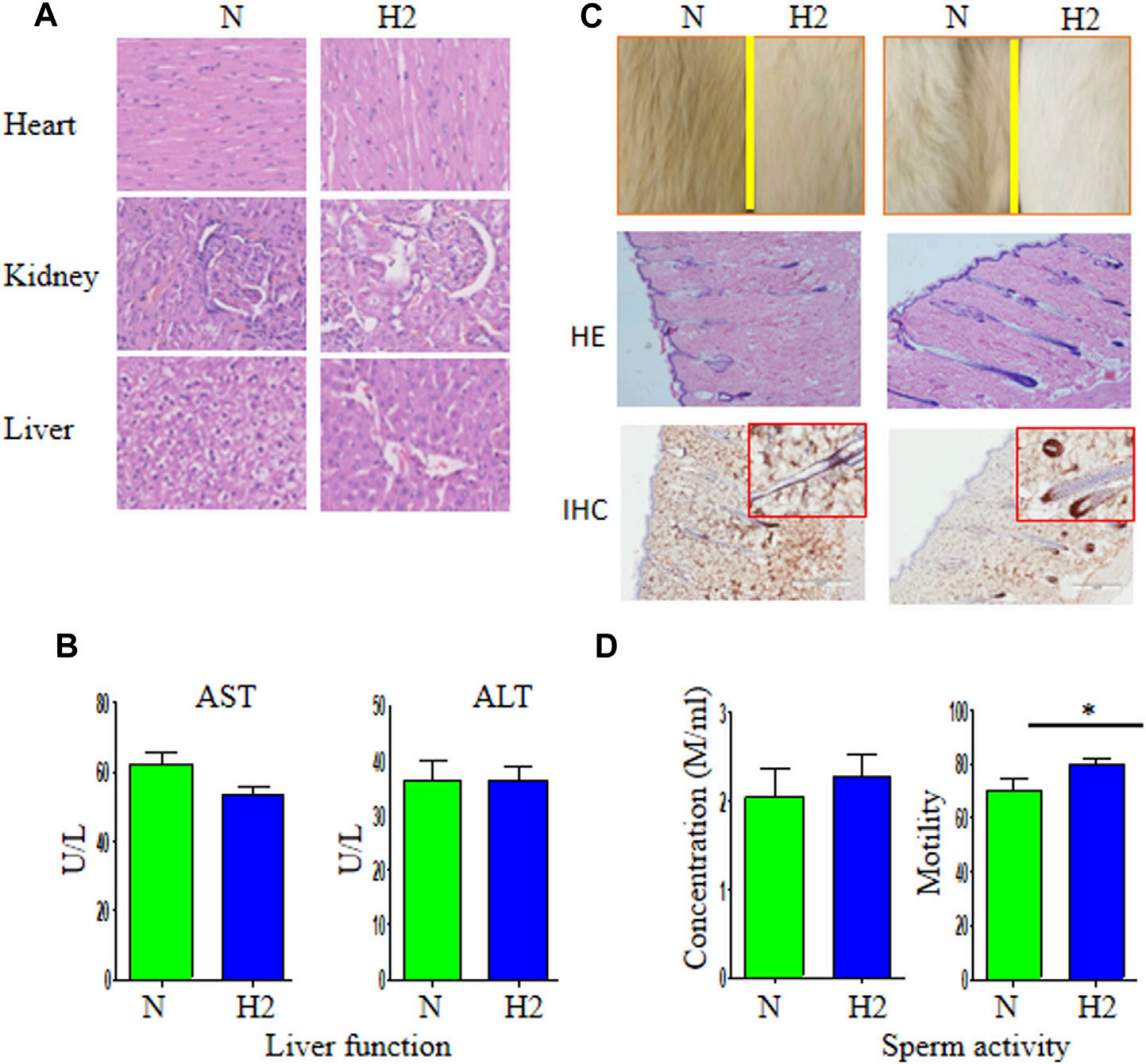
The safety of long-term use of H_2_-rich water and its effects on quality of life of rats. **(A)** Healthy individuals were given regular water (N) and H_2_-rich water (H_2_) for 9 months and sacrificed for the detection of pathologic morphology of heart, kidney, and liver. **(B)** The levels of AST and ALT in two groups. **(C)** The hair and fur were observed in these healthy individuals. Hematoxylin and eosin staining (HE) and immunohistochemistry (Ki67) showed more hair shaft (the fiber) and hair bulb (the root) in rats receiving H_2_-rich water than those without drinking H_2_-rich water. **(D)** The concentration and motility of sperm of these rats. *p < 0.05. HE, IHC × 200.

There was no instances of TAA- or drug-related mortality during all experiments. The first experiment mainly focused on the safety of use of H_2_-rich water. 9 months use of H_2_-rich water did not cause any pathological changes of internal organs including heart, liver and kidney compared with the control group (Figure 1A). No significant differences were observed in the levels of AST or ALT between two groups (Figure 1B). However, we observed two exciting events during this period: the hair of rats drinking H_2_-rich water became whiter and more lustrous compared with those not drinking H_2_-rich water (Figure 1C); the former had higher sperm motility than the later (Figure 1D). In additon, HE and IHC staining revealed denser hair follicles and higher proliferative activity in the former rats than in the later rats (Figure 1C).

### Hydrogen-Rich Water Inhibit Cholangiofibrosis Formation Induced by TAA

**FIGURE 2:**
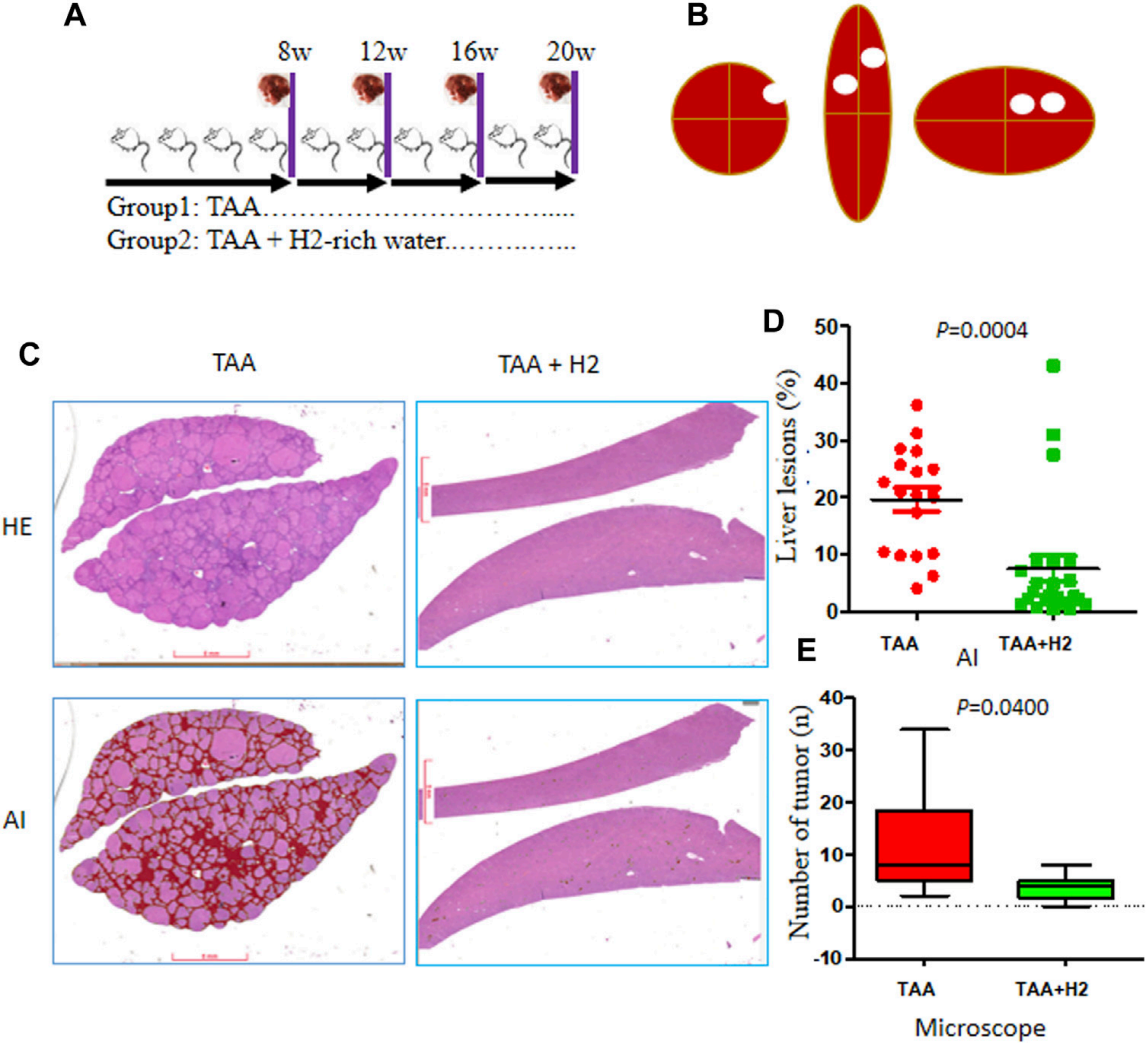
The morbidity of intrahepatic lesions in each group at 20 weeks. **(A)** Three (TAA and TAA + H_2_) of rats were sacrificed and examined at 8, 12, 16, and 20 weeks. **(B)** White nodules on the surface and within the liver were counted. **(C)** Automated histological classification was developed to evaluated liver lesions based on convolutional neural networks (CNNs). Representative whole slide images of liver sections from the two groups were presented (H&E: upper; AI: lower). AI, artificial intelligence. **(D)** The ratio of liver lesions assessed by the AI algorithm in the two groups. **(E)** The number of microscopic cholangiofibrosis counted under optical microscope in two groups.

Sequential harvesting of the liver is shown in Figure 2A. Tumor nodules on the liver surface and on each liver section were thoroughly counted and tabulated (Figure 2B). All of the rats (9/9, 100%) in the TAA group and 28.6% (2/7) of rats in the TAA + H_2_ group developed visible white nodules in the liver. Overall, the average number of macroscopic white nodules in the TAA group was 7.4 ± 5.7, which was significantly higher than that observed in the TAA + H_2_ (1.3 ± 2.6) groups.

The most obvious and important characteristics of the liver lesions were as follows: fibrosis (characterized by α-SMA expression) (Supplementary Figures S2A,E) and cholangiofibrosis (characterized by CK7 and CK19 overexpression) (Supplementary Figures S2B–D). Here, we developed an automated lesion detection framework based on deep learning convolutional neural networks (CNNs). This AI algorithm clearly showed the location and quantities of liver lesions (red) in WSIs (Figure 2C). The ratio of fibrosis lesions recognized by AI was 19.6% ± 9.01 in the TAA group, which was significantly higher than those of the TAA + H_2_ (7.53% ± 11.0) groups (Figure 2D). We also calculated microscopic “intestinal-type” CCAs under optical microscope. All of the rats (100%, 9/9) in the TAA group developed microscopic cholangiofibrosis, corresponding to a microscopic average of 12 ± 10.1 per rat. Strikingly, only 57.1% (4/7) of the rats in the TAA + H_2_ group developed microscopic cholangiofibrosis at a rate of 2.9 ± 5.4 lesions per rat, whereas the other 42.9% of rats did not harbor any detectable cholangiofibrosis. The incidence of cholangiofibrosis in the TAA group was significantly higher than those of the TAA + H_2_ (*p* = 0.0487) group (Figure 2E). These data provide first-hand evidence that H_2_–rich water could inhibit the development of cholangiofibrosis.

### H_2_-Rich Water Prevents the Progression of TAA-Induced Fibrosis and Cholangiofibrosis

**FIGURE 3:**
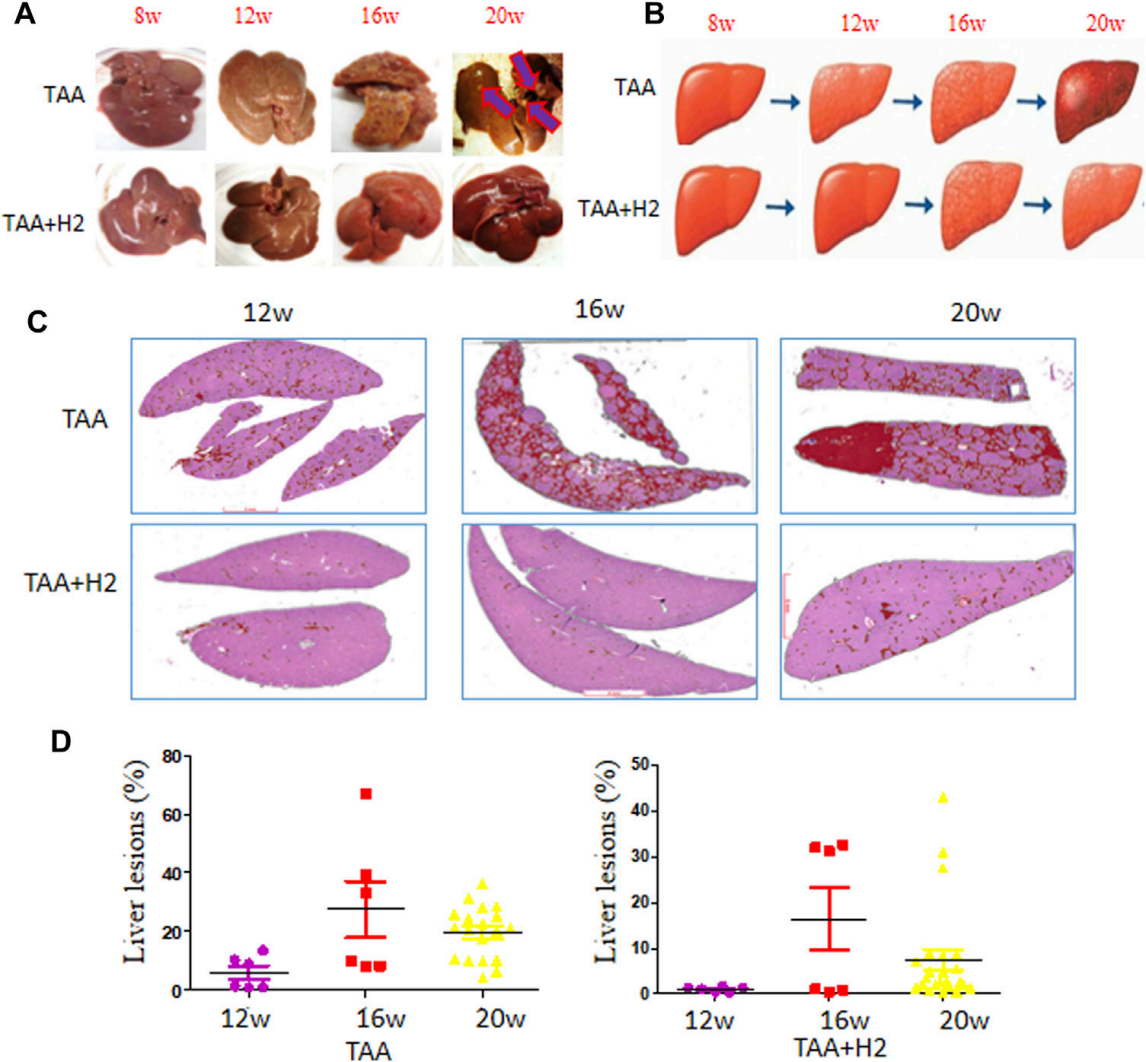
Microscopic profiles of the liver lesions during TAA induction with or without H_2_-rich water administration. **(A)** Representative gross images of the livers at the indicated time points. **(B)** Schematic diagram of the liver status treated with TAA with or without hydrogen-rich water supplementation. **(C)** Representative images of cholangiolar lesions of the livers at the indicated time points analyzed by the AI algorithm. **(D)** The ratio of cholangiolar lesions of the livers assessed by the AI algorithm on representative slides at the indicated time points.

Sequential observation showed that the morphology of the liver induced by TAA began to deteriorate at 12 weeks and dramatically deteriorated at 16 and 20 weeks in the TAA group. In the TAA + H_2_ group, the liver morphology began to deteriorate at 16 weeks and only worsened in 28.6% of cases (2/7) at 20 weeks (Figures 3A,B).

We analyzed microscopic liver lesions by our AI algorithm that arose from TAA induction in the presence or absence of hydrogen-rich water treatment. The ratio of liver lesions increased sharply along with time in the TAA group (Figure 3C upper and Figure 3D left), whereas in the TAA + H_2_ group, the ratio only increased at 16 weeks, after which they decreased to low levels (Figure 3C lower and Figure 3D right). These data further confirm that H_2_ treatment delays the progression from fibrosis to cholangiofibrosis.

### Hydrogen Decreases Glycolysis and Interrupts Cross-Talk Between Proliferating Bile Duct Cells and the Microenvironment

**FIGURE 4:**
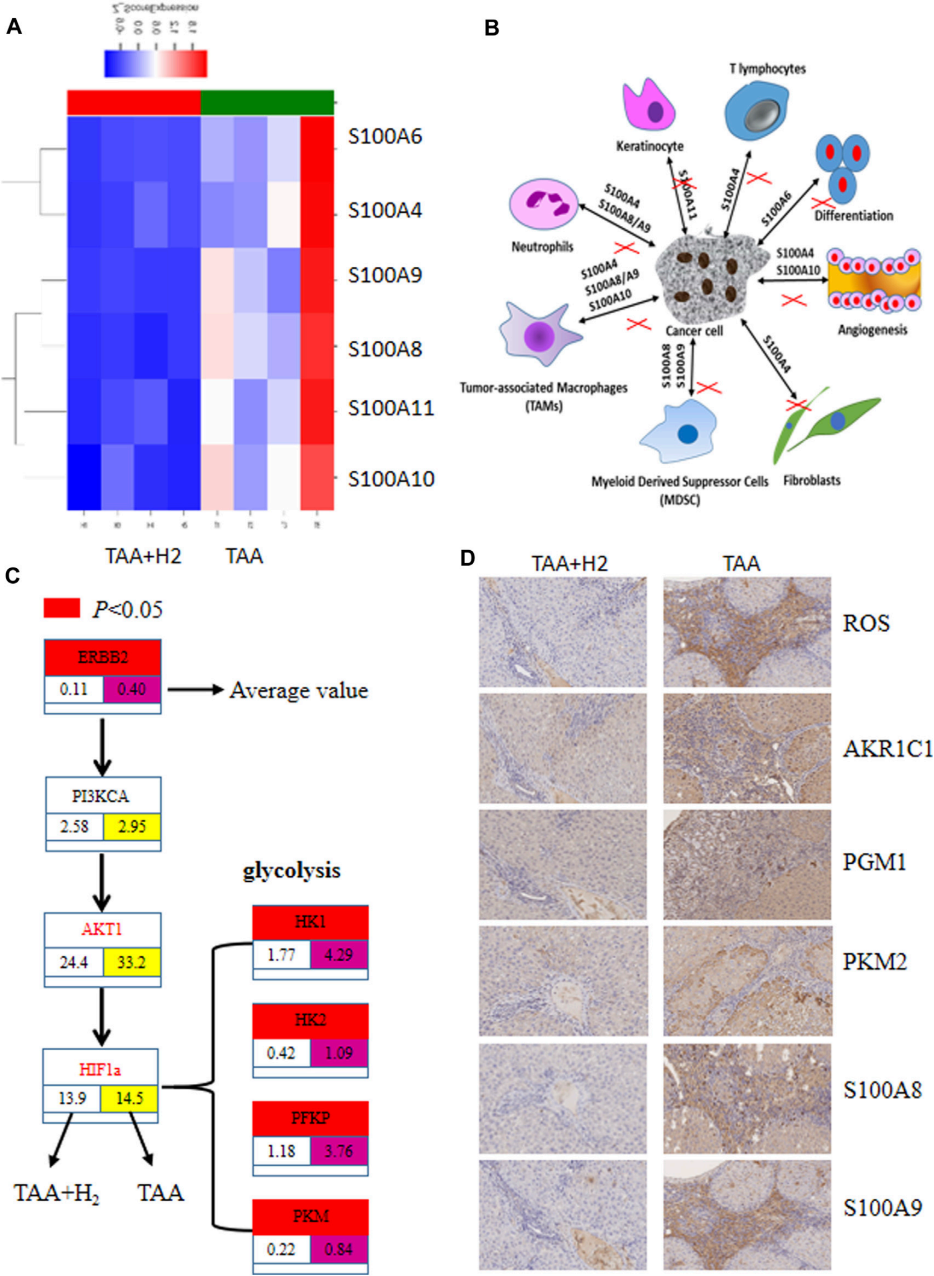
RNA-seq analysis revealed differential gene expression between the livers from the TAA and TAA + H_2_ groups. **(A)** Four livers from the TAA group and four livers from the TAA + H_2_ group were subjected to RNA-seq analysis, and multiple members of the S100 gene family were highly expressed in the TAA group but showed low expression in the TAA + H_2_ group. **(B)** Schematic diagram of the interaction between cancer cells and stroma cells which can be blocked by H_2_. **(C)** ERBB2 and rate-limiting enzymes in glycolysis were significantly decreased by H_2_ treatment compared with no H_2_ treatment. **(D)** Immunohistochemistry was used to detect the expression of ROS, AKR1C1, PGM1, PKM2, S100A8, and S100A9 on the liver samples from the TAA group and the TAA + H_2_ group. IHC ×100.

Next, we used RNA-seq analysis to analyze the changes that occur when TAA-induced cholangiofibrosis are treated with H_2_-rich water. Unsurprisingly, we observed significant differences in the gene expression profiles between these two groups (Supplementary Figures S3A,B). Multiple signaling pathways were substantially affected by H_2_ treatment (Supplementary Figures S3C,D), including genes in pathways associated with cell adhesion and inflammation. Multiple members of the S100 family were increased by TAA administration, and hydrogen-rich water down-regulated the expression of these genes three- to 5-fold (Figures 4A,B). Further analysis showed that ERBB2 was down-regulated in the TAA + H_2_ group. In addition, we found that the three rate-limiting enzymes in glycolysis, hexokinase (HK), phosphofructokinase (PFK), and pyruvate kinase, were down-regulated two-to four-fold in rats that received hydrogen-rich water (Figure 4C). Immunohistochemistry showed that, except ROS, H_2_ also visibly decreased the expression several glycolysis-associated proteins: AKR1C1, PKM2, PGM1 and two members of S100 family: S100A8 and S100A9 in the liver lesions (Figure 4D).

### Hydrogen Interrupts TAA-Initiated Cholangiofibrosis After Leaving the Sources of Pollution

**FIGURE 5:**
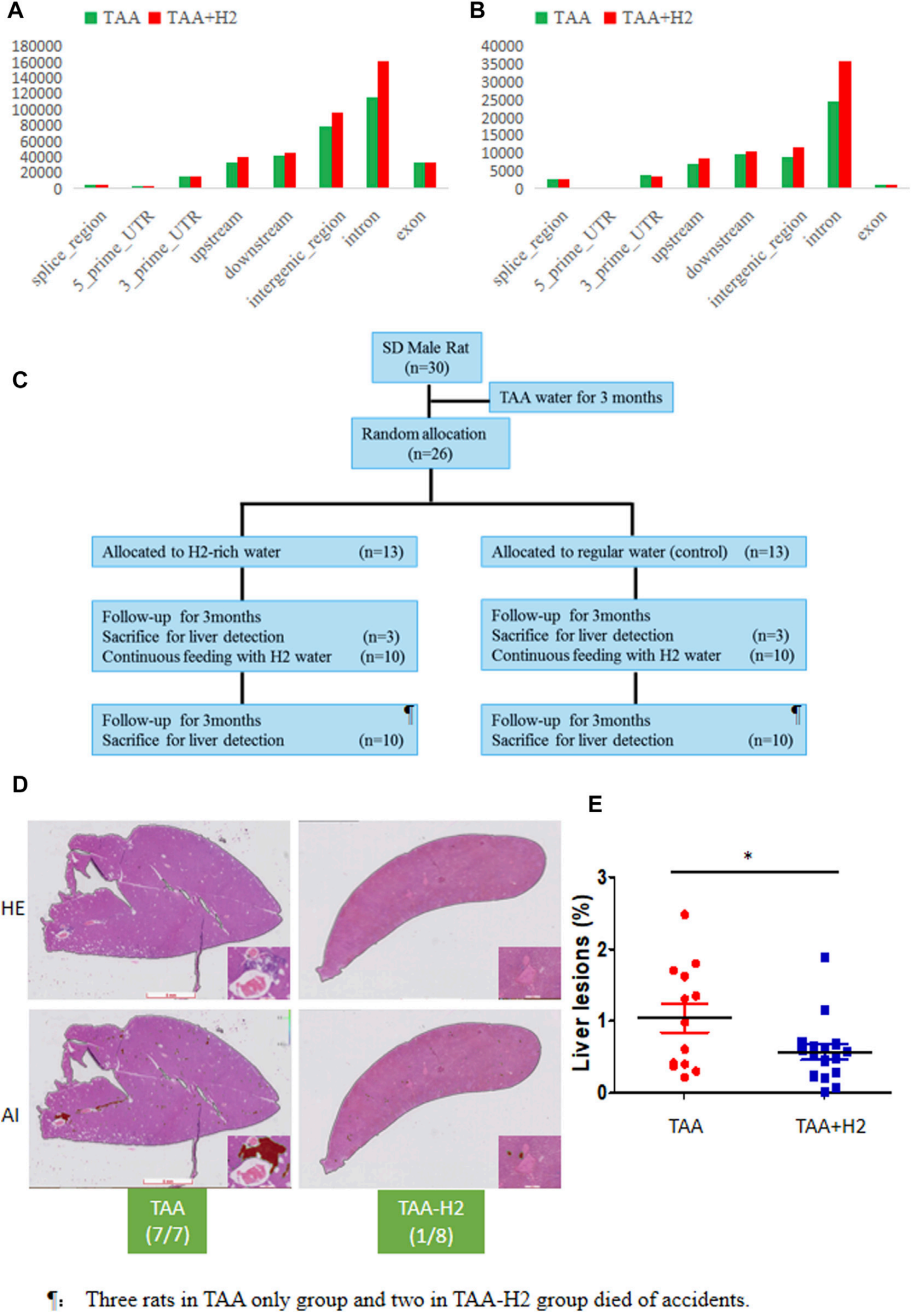
Long-term use of H_2_-rich water interrupts the TAA-initiated cholangiofibrosis. **(A, B)** RNA-seq showed the number of SNP **(A)** and deletion/insertion **(B)** in the liver lesions from the TAA and TAA + H_2_ groups. **(B)** An experimental flow chart was designed to assess whether molecular hydrogen could interrupt the procession of cholangiofibrosis. **(C)** All rats developed cholangiofibrosis or suspicious cholangiofibrosis in TAA-pretreated rats without following treatment, while only one rat developed cholangiofibrosis if treated with H_2_-rich water. **(D)** The ratio of liver lesions determined by the AI algorithm in two groups. *p < 0.05.

Although H_2_ administration reduced the incidence of cholangiofibrosis in TAA-induced cholangiofibrosis models, the ratio of mutation was not affected by H_2_ intervention analyzed by RNA-sequencing of the liver lesions (Figures 5A,B). Given the fact that high mortality of certain tumors in first-generation of migration can be reduced in subsequent generations (Mangtani et al., 2010), we mimicked this situation by removing TAA after 3 months of feeding SD rats without any intervention (Figure 5C). If left untreated, 100% rats (7/7) developed microscopic cholangiofibrosis in the 6th month. However, only 12.5% (1/8) developed microscopic cholangiofibrosis if these rats were administrated with H_2_-rich water for 6 months. The AI algorithm also confirmed that the ratio of Alesion/Atissue in TAA-pretreated rats was significantly reduced in those with H_2_ intervention (Figures 5D,E).

### Hydrogen Alters the Gut Microbiota Composition

**FIGURE 6:**
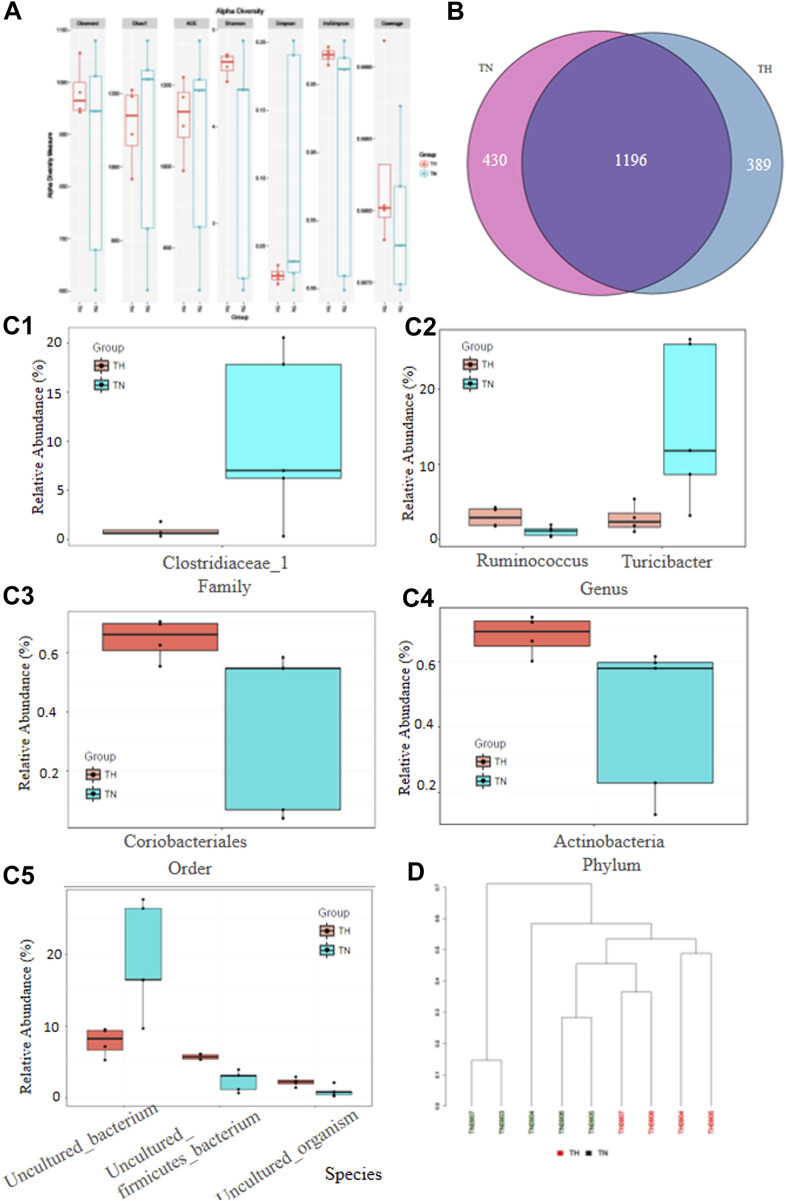
Hydrogen alters the gut microbiota composition. **(A)** Whole-genome sequencing was performed for fecal samples from the TAA-pretreated rats followed with or without H_2_ intervention and alphaDiversity between two groups was presented. **(B)** The average number of the gut microbiota in each group and the intersection number of the two groups. **(C)** The relative abundance of different taxonomic levels of bacterial strains in the two groups, including family (clostridiaceae_1) (C1), genus (ruminococcus and turicibacter), order (coriobacteriales) (C3), phylum (actinobacteria) (C4), and species (uncultured firmicutes_bacterium) (C5). **(D)** Cluster analysis of the nine rats treated with or without H_2_-rich water.

Given the power of H_2_-rich water in relieving constipation, we investigated how H_2_ affected the composition of the gut microbiota. To characterize the effects of H_2_ on the gut microbiome, we performed whole-genome sequencing of fecal samples from the second experiment. Finally, only five samples from TAA-regular water group and four from TAA-H_2_ group were available for analysis. Even in this small cohort, we also observed a remarkable difference of alphaDiversity between this two groups (Figure 6A). Totally, the average number of the gut microbiota was 1,626 in TAA-regular water group and 1,589 in TAA-H_2_ group, and the intersection number was 1,196 (Figure 6B). H_2_ treatment for 5 months resulted in significant alterations in the relative abundance of different taxonomic levels of bacterial strains, including family (clostridiaceae_1), genus (ruminococcus and turicibacter), order (coriobacteriales), phylum (actinobacteria), and species (uncultured firmicutes_bacterium) (Figure 6C). Cluster analysis clearly separated all rats into different groups (Figure 6D).

## Discussion

Cancer mortality was the second leading cause of mortality worldwide in 2012 and could eventually become the number one cause of death if changes are not made (Chen et al., 2016). The conditions in China are particularly poor because of severe changes in lifestyle choices and deterioration of the environment as a result of rapid industrialization and urbanization (Bode et al., 2016). The concept of “precision medicine” has attracted almost all public and private research investments. However, the growing costs of new cancer drug treatments and molecular detection methods are unsustainable for developing countries or districts. The most attractive solution to this potential health and economic catastrophe is to “prevent the preventable” (Albini et al., 2016). In addition to avoiding risk factors and maintaining a healthy lifestyle, compounds that prevent cancer initiation could offer an effective preventive approach to decreasing cancer risk. However, funding for cancer prevention constitutes less than 3% of all cancer research funding (Albini et al., 2016), and this figure is even lower in developing countries. These funds are not enough to develop effective cancer preventive drugs with minimal toxicity. Existing drugs, such as aspirin, metformin, and tamoxifen, could be useful alternatives. Very recently, individuals between the ages of 40 and 69 years with a normal bleeding risk have been shown to benefit from consuming aspirin daily for the primary prevention of cardiovascular disease and colorectal cancer (Dehmer et al., 2015). However, the increased risk for gastrointestinal (GI) and cerebral hemorrhages render the prophylactic use of aspirin unacceptable in certain settings. Here, we demonstrate that molecular hydrogen is an effective and safe modality for cancer prevention.

Cholangiocarcinoma (CCA) is a disease entity comprising diverse epithelial tumours united by late diagnosis and poor outcomes (Rizvi et al., 2018). Cholangiofibrosis is a controversial intrahepatic cholangial lesion defined by proliferative, metaplastic, and inflammatory components, which precedes the development of cholangiocarcinoma in chemically treated rats (Hickling et al., 2010; Adams et al., 2011). In these rodent models, the cholangiolar epithelium displays unqique morphological features including dilated glands lined by hyperbasophilic and metaplastic epithelium (intestinal metaplasia), which was called “cholangiocarcinoma, intestinal type” as previously proposed (Yeh et al., 2004; Wang et al., 2018). This model is ideal to test the efficacy of cholangiofibrosis preceding to tumor initation. After treating this model with molecular hydrogen for a 5 month period, we evaluated the preventive efficiency of these treatments in two aspects: liver lesions recognized by our developed AI algorithm and microscopic cholangiofibrosis. As expected, the ratio of liver lesions in rats that received H_2_-rich water was much better than those from rats that did not receive treatment. Moreover, we found that the number of visible and microscopic cholangiofibrosis in the liver decreased after H_2_-rich water treatment. Collectively, these data confirm the preventive effect of H_2_-rich water in CCA carcinogenesis. Importantly, we did not observe any side effects of long-term use of H_2_-rich water. Whether the consumption of H_2_-rich water could be used to prevent cancer in high-risk populations will require further investigation.

Several studies proposed that molecular hydrogen acts as an antioxidant by quenching reactive oxygen species (ROS). Subsequent studies showed that molecular hydrogen suppresses multiple signaling pathways and downstream targets in inflammation (Itoh et al., 2011; Ichihara et al., 2015). Hydrogen also modifies the activity and expression levels of signaling molecules, including NF-kB p65 or NF-kB, STAT3, GSK-3ß, JNK, Ras, ERK, and JNK. In our study, we found that hydrogen inhibits ERBB2 overexpression, which is a driver that accelerates CCA tumorigenesis. A unique characteristic of cancer cells is the generation of energy and anabolic metabolism through glycolysis, along with high expression levels of rate-limiting enzymes catalyzing the first (HK), middle (PFK), and last (PKM) steps of glycolysis. Hydrogen has the ability to attenuate this metabolic process, indicating that it may inhibit tumorigenesis through the modulation of glycolysis. Moreover, cholangiocarcinoma is characterized by the infiltration of inflammatory cells and uncontrolled hyperplasia of the tumor stroma. Our analysis showed that the expression levels of the members of the S100 family (S100A4, −6, −8, −9, −10, and −11) were dramatically decreased when H_2_-rich water was administered long-term. S100 proteins facilitate communication between proliferative cells and stromal cells, including fibroblasts, endothelial cells, and inflammatory cells (tumor-associated macrophages, myeloid-derived suppressor cells, T lymphocytes, and neutrophils) (Chen et al., 2014). Long-term use of hydrogen-rich water appears to disrupt this cancer-stroma interaction.

Cancer interception involves the interruption of the process of cancer development during the early stages to slow or even reverse the growth of a tumor mass from transformed foci (Albini et al., 2016). In our study, we aimed to further evaluate the interceptive strategy of molecular hydrogen in reducing cancer progression. To test this question, we have begun treating rats with TAA for three months, after which we will treat the rats with H_2_-rich water. The final results were positive. When administrated with H_2_-rich water, no rats developed cholangiofibrosis, the ratio of liver lesions dramatically decreased, indicating that molecular hydrogen has the potential in interrupting the process of cancer development. This result provided a solution for those individuals exposure to contaminants at their earlier ages.

To investigate why long-term use of H_2_-rich water can relieve constipation and prevent cancer initiation, we performed whole-genome shotgun sequencing of fecal samples obtained from TAA-pretreated rats with or without following intervention of H_2_-rich water. Hydrogen did lead to significant changes of the composition of the gut microbiota. Use of hydrogen decreased the abundance of clostridiaceae_1, which correlated with the inflammation caused by antibiotic treatment (Unno et al., 2015). Ruminococcus, known for fermenting polysaccharide into short chain fatty acids, was decreased, while turicibacter, associated with elevated inflammation in the obese status, was increased by use of hydrogen (Jiao et al., 2018b). The members of Coriobacteriaceae are supposed be indicators of a healthy GI microbiota (Krogius-Kurikka et al., 2009). Hydrogen could upregulate the abundance of actinobactteria, which demonstrates beneficial effects in many pathological conditions (Binda et al., 2018). Our findings supported for the notion that altered gut microbiota mediates the effects of hydrogen on interrupting tumor progression and improving quality of life. It is not clear whether these changes of gut microbiota are related with the host’s physiological functions, and further studies are necessary for understanding the roles of each altered taxon in maintaining homeostasis.

In conclusion, our data show for the first time that long-term use of H_2_-rich water protects against liver injury and significantly decreases the morbidity of chemically induced cholangiofibrosis. Moreover, H_2_-rich water improved the QOL of rats. These results indicate that long-term use of H_2_-rich water is safe and effective. The mRNA levels of key proteins in glycolysis and the S100 family were dramatically decreased by hydrogen consumption, which could be due to molecular hydrogen antioxidant activity. Our results also showed that the use of hydrogen inhibited potential pathogenic bacteria during the initiation of cholangiofibrosis. However, all our findings are descriptive evidicence, which cannot disclose the direct mechanisms and direct targeted organs of H_2_. More investigation is needed to determine the underlying mechanisms of hydrogen-dependent in cancer prevention and to explore the potential clinical value.

## Data Availability

The datasets presented in this study can be found in online repositories. The names of the repository/repositories and accession number(s) can be found below: NCBI SRA BioProject, accession no: PRJNA728140.
