# Supplementary figures and images for: The Preventive Role of Hydrogen-Rich Water in Thioacetamide-Induced Cholangiofibrosis in Rat Assessed by Automated Histological Classification
Source: Front Pharmacol. 2021 Aug 20;12:632045. doi: 10.3389/fphar.2021.632045 (PMC8417776; doi:10.3389/fphar.2021.632045)

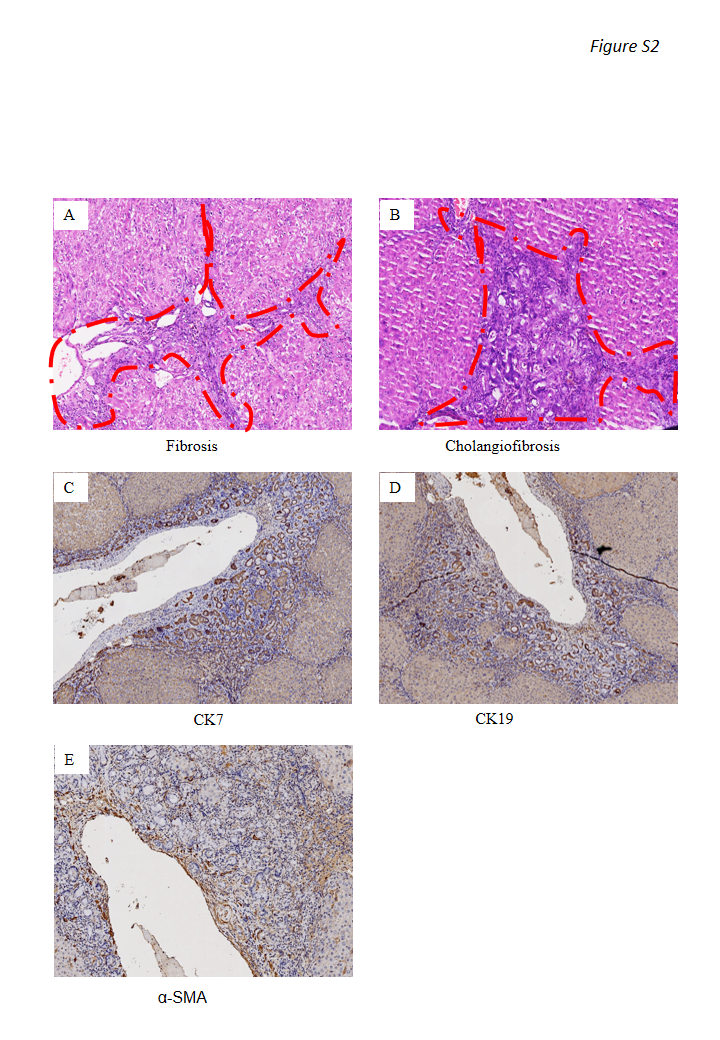

Supplement: Supplementary file 1 [file Image3.TIF]

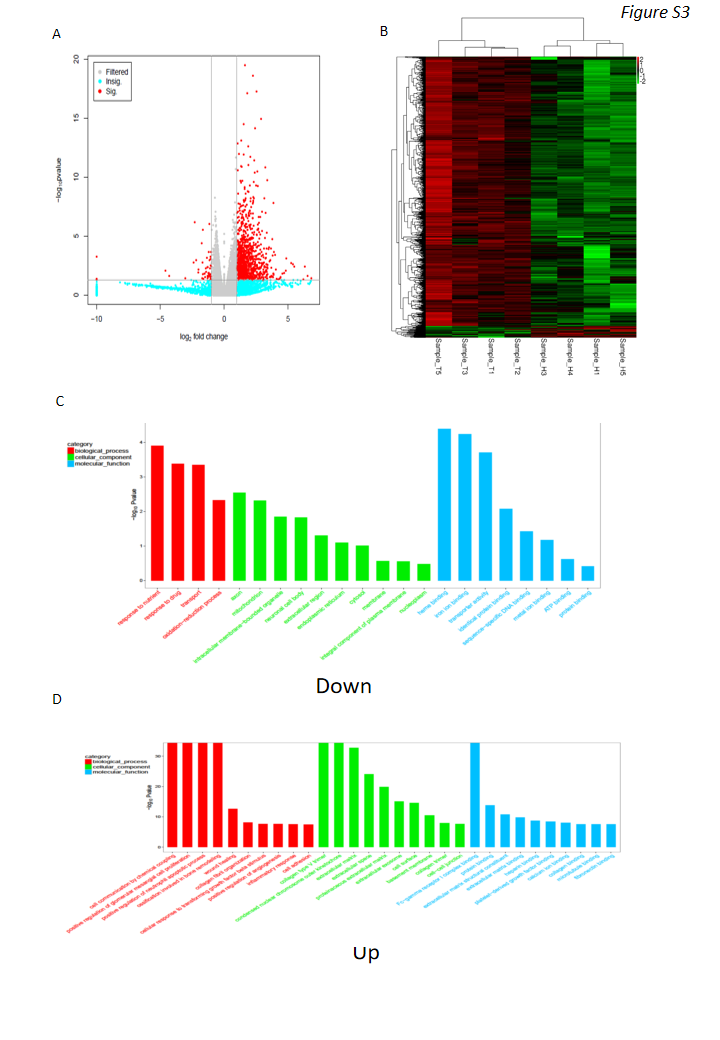

Supplement: Supplementary file 2 [file Image2.TIF]

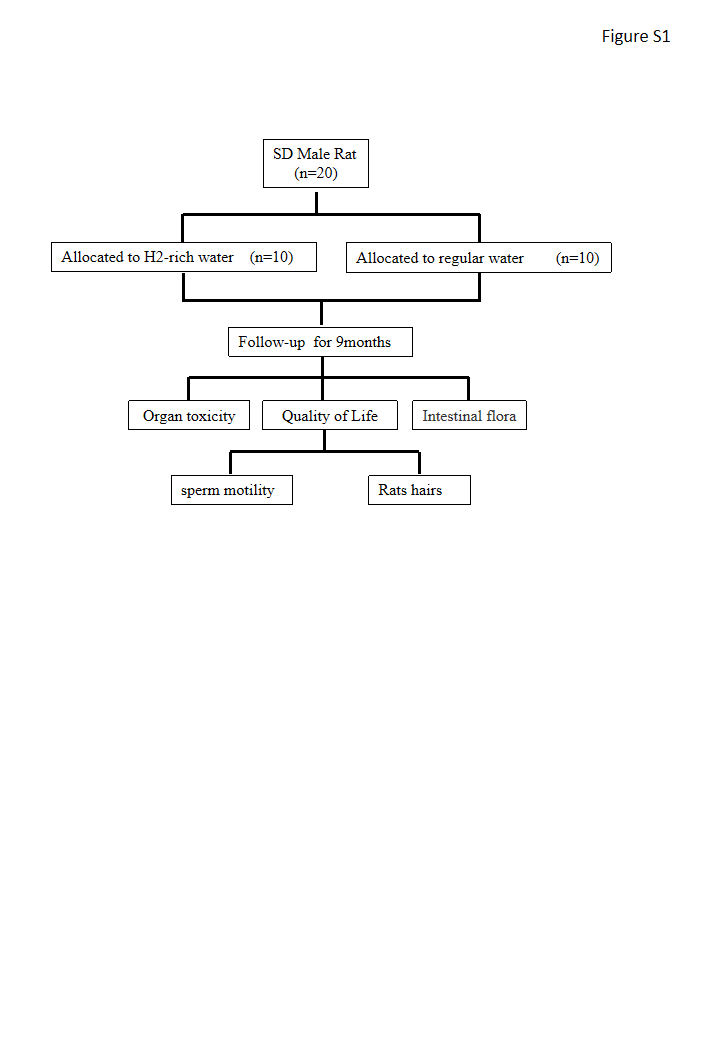

Supplement: Supplementary file 3 [file Image1.TIF]
